# Akt/mTOR Targeting Activity of Resveratrol Derivatives in Non-Small Lung Cancer

**DOI:** 10.3390/molecules27238268

**Published:** 2022-11-27

**Authors:** Bhurichaya Innets, Sunisa Thongsom, Korrakod Petsri, Satapat Racha, Masashi Yokoya, Sohsuke Moriue, Chatchai Chaotham, Pithi Chanvorachote

**Affiliations:** 1 Center of Excellence in Cancer Cell and Molecular Biology, Faculty of Pharmaceutical Sciences, Chulalongkorn University, Bangkok 10330, Thailand; 2 Department of Pharmacology and Physiology, Faculty of Pharmaceutical Sciences, Chulalongkorn University, Bangkok 10330, Thailand; 3 Doctor of Philosophy Program in Physiology, Graduate School, Chulalongkorn University, Bangkok 10330, Thailand; 4 Interdisciplinary Program in Pharmacology, Graduate School, Chulalongkorn University, Bangkok 10330, Thailand; 5 Department of Pharmaceutical Chemistry, Meiji Pharmaceutical University, 2-522-1, Noshio, Kiyose, Tokyo 204-8588, Japan; 6 Department of Biochemistry and Microbiology, Faculty of Pharmaceutical Sciences, Chulalongkorn University, Bangkok 10330, Thailand

**Keywords:** Akt/mTOR targeting, non-small lung cancer, resveratrol derivatives

## Abstract

The Akt-mTOR signal is important for the survival and proliferation of cancer cells and has become an interesting drug target. In this study, five resveratrol derivatives were evaluated for anticancer activity and Akt/mTOR targeting activity in non-small lung cancer cell lines. The effects of resveratrol derivatives on cell proliferation were assessed by 2,5-diphenyl-2H-tetrazolium bromide (MTT) assay, nucleus staining, and colony formation assay. Furthermore, the effect of resveratrol derivatives on proliferation-related protein expression was analyzed by immunofluorescence and Western blotting. For the structure–activity relationship (SAR), results reveal that two derivatives of resveratrol which are 4,4′-(ethane-1,2-diyl) bis(2-methoxyphenol) (RD2) and the 4-(3-hydroxy-4-methoxyphenethyl)-2-methoxyphenol (RD3) had very similar structures but exerted different cytotoxicity. The IC_50_ of RD2 and RD3 were 108.6 ± 10.82 and more than 200 µM in the A549 cell line and 103.5 ± 6.08 and more than 200 µM in H23 cells, respectively. RD2 inhibited cell proliferation and induced apoptosis when compared with the control, while RD3 caused minimal effects. Cells treated with RD2 exhibited apoptotic nuclei in a concomitant with the reduction of cellular *p*-Akt and *p*-mTOR. RD3 had minimal effects on such proteins. According to these results, molecular docking analysis revealed a high-affinity interaction between RD2 and an Akt molecule at the ATP-binding and the allosteric sites, indicating this RD2 as a potential Akt inhibitor. This study provides useful information of resveratrol derivatives RD2 for treating lung cancer via Akt/mTOR inhibition.

## 1. Introduction

Lung cancer is considered one of the important cancers according to its high rate of mortality. In addition, it was found that new cases of lung cancer are relatively high in comparison to other human cancers [1]. Non-small Lung Cancer (NSCLC) is the most common type of lung cancer, and this disease is frequently found at an advanced stage (III-IV) at the time of first detection [2]. The treatment for NSCLC is surgery, radiotherapy, chemotherapy, immunotherapy, and molecularly targeted therapy [3]. High incidence of chemotherapeutic resistance in NSCLC is one factor causing failure of the treatment and cancer recurrence [4]. Currently, drugs, as well as novel compounds possessing targeted action to the cancer cells, have gained interest as potential means to improve the clinical outcome.

It was demonstrated in many studies that the phosphatidylinositol 3-kinase (PI3K)/Protein kinase B (Akt)/mechanistic target of rapamycin (mTOR) pathway is a promising target for cancer therapy. This pathway is known as the regulator of cancer cell survival and growth. The augmented activation or amplification of PI3K/Akt/mTOR pathway leads to the inhibition of apoptosis, increased rate of cell proliferation, and induction of metastasis. It has been found that several PI3K/Akt/mTOR targeted therapies such as pictilisib (PI3K inhibitor) and perifosine (Akt inhibitor) are undergoing clinical trials as treatments for lung cancer [5]. Although current cancer therapy has achieved improvements, the survival of lung cancer patients remains disappointing [6]. Development of novel treatment options or novel potential drugs targeting cancer progression has important practical needs. 

Resveratrol, a 3,5,4′-trihydroxystilbene, has several pharmacological activities including anticancer activity against lung cancer [7]. Resveratrol was demonstrated to work as a chemo-preventive agent through its proapoptotic, anti-proliferation, and anti-inflammation effects. The anticancer activity of this compound involves its property to induce cell apoptosis and inhibit cancer initiation and cancer progression [8,9,10,11]. Resveratrol was demonstrated to inhibit cell proliferation and reduction of the pro-survival pathway in a lung cancer cell model [12]. In NSCLC cells, resveratrol was demonstrated in A549 cells to induce autophagy and apoptosis via the inhibition of the NGFR-AMPK-mTOR signaling pathway [13]. In addition, resveratrol was shown to inhibit Akt/mTOR and activate the p38-MAPK pathway in A549 cells [14]. 

The structure of the drug is critical for the drug interaction with specific molecules of drug targets. The modification of the compound structure may lead to the alteration of the compound-drug affinity and the binding stability which directly controls the drug action. Hence, studies of derivatives which are structurally modified from the potential lead compound resveratrol have constituted the basis for the development of new analogues and may improve its effect on cancer cells [15]. As resveratrol has a relatively simple structure and contains several functional groups that could be modified including benzene diol groups, hydroxyphenyl, and aromatic rings, we synthesized the new analogues of resveratrol derivatives and tested for anti-cancer activity and their effect in targeting Akt/mTOR signaling pathway as well as the effect of chemical structure modification on such a compound action. 

## 2. Result

### 2.1. Resveratrol Derivative Compounds

**Figure 1:**
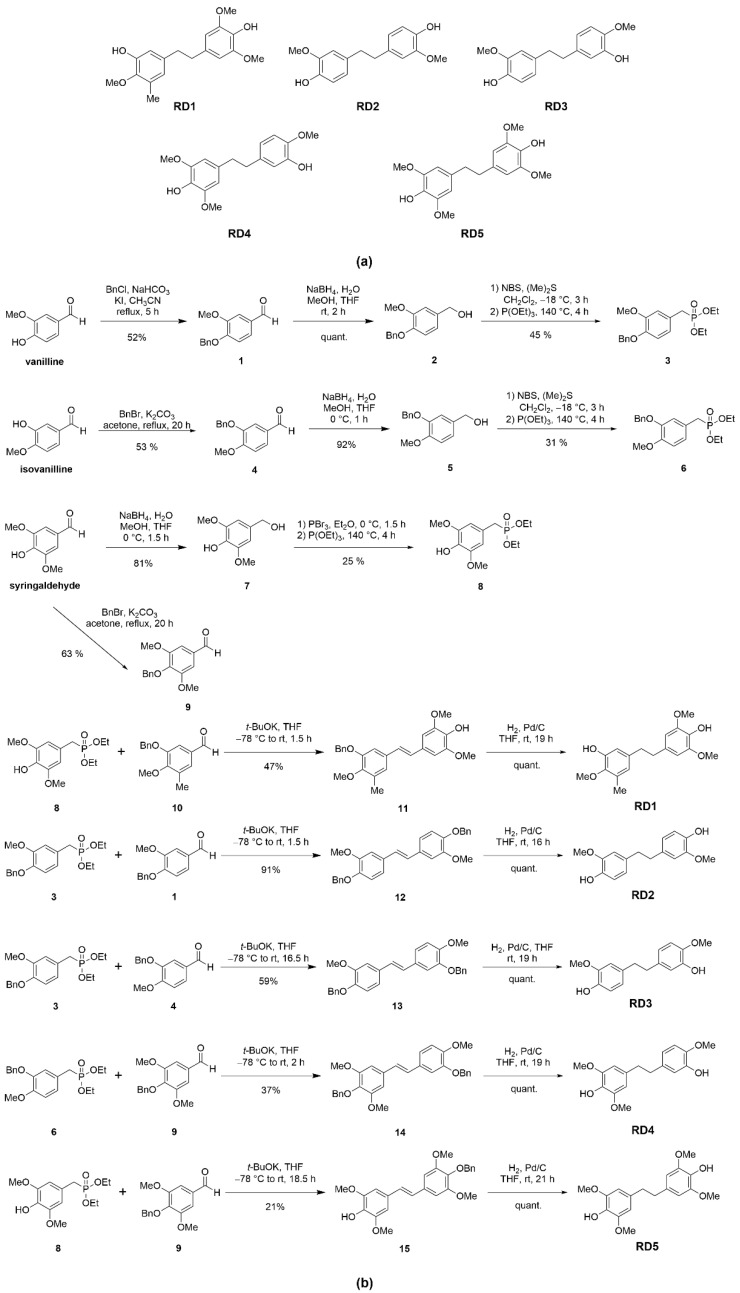
Synthesis and characterization of resveratrol derivatives. (**a**) Structure of five resveratrol derivatives’ compounds (RD1-RD5). (**b**) The synthesis method of five resveratrol derivatives’ compounds.

Resveratrol derivatives were obtained from chemical synthesis starting from aromatic aldehydes (vanillin and isovanillin) as described in Materials and Methods. We aimed to gain five novel resveratrol derivatives, which are 5-(4-hydroxy-3,5-dimethoxyphenethyl)-2-methoxy-3-methylphenol (RD1), 4,4′-(ethane-1,2-diyl)bis(2-methoxyphenol) (RD2), 4-(3-hydroxy-4-methoxyphenethyl)-2-methoxyphenol (RD3), 4-(3-hydroxy-4-methoxyphenethyl)-2,6-dimethoxyphenol (RD4), and 4,4′-(ethane-1,2-diyl)bis(2,6-dimethoxyphenol) (RD5) (Figure 1a).

### 2.2. Cytotoxicity and Inhibition of Colony Forming Capacity of Resveratrol Derivatives

To investigate the anti-cancer potentials of resveratrol derivatives RD1, RD2, RD3, RD4, and RD5 on lung cancer cells, we determined the cytotoxic profile of resveratrol derivatives (RD1-RD5) and resveratrol in several lines of NSCLC cells namely A549, H23, and H460 cells.

**Figure 2:**
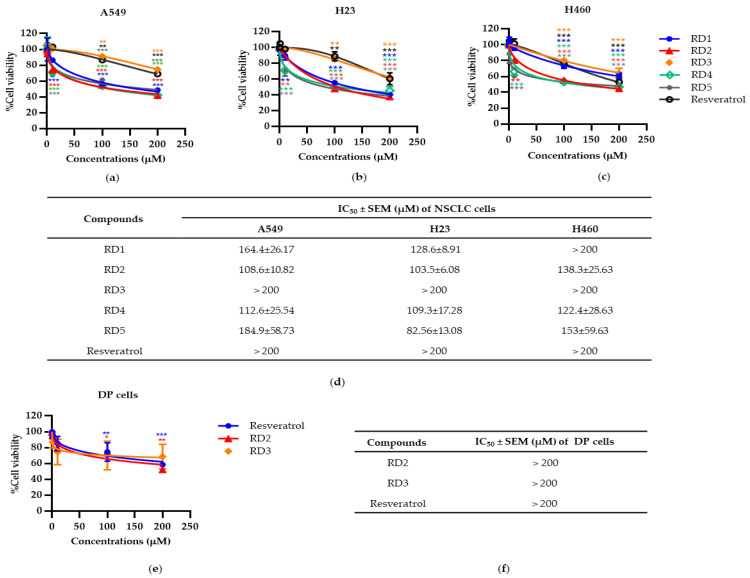
Resveratrol derivatives (RD1-RD5) and resveratrol decreases cell viability in NSCLC cells (A549, H23, and H460) and the normal cell line (DP cells). (**a**–**c**) MTT assay was used to evaluate cell viability after treatment with various concentrations of RD1-RD5 and resveratrol (0–200 mM) for 24 h in A549, H23, and H460 cells, respectively. (**d**) The IC_50_ of RD1-5 and resveratrol against A549, H23, and H460 cells was calculated from MTT assay by comparison with an untreated control. (**e**) MTT assay was used to evaluate cell viability after treatment with various concentrations of RD2, RD3, and resveratrol (0–200 mM) for 24 h in DP cells, respectively. (**f**) The IC_50_ of RD2, RD3, and resveratrol against DP cells was calculated from MTT assay by comparison with an untreated control. Data are represented as the mean ± SEM. * *p* < 0.05, ** *p* < 0.01, *** *p* < 0.001 when compared with the control group.

NSCLC cells were cultured overnight and treated with various concentrations (0–200 µM) of resveratrol derivatives (RD1-RD5) and resveratrol for 24 h, and then cell viability was analyzed by the 3-(4, 5-dimethylthiazolyl-2)-2, 5-diphenyltetrazolium bromide (MTT) assay to evaluate the percentage of cell viability. All of the data were calculated on the basis of the results of three replicated samples. The results revealed that RD1-RD5 compounds shown potential cytotoxic effects. Among them, we found that RD2, which had a similar structure to RD3 (Figure 1a), exerted a dramatically different cytotoxic effect on lung cancer cells. The difference of the structure at C-4 was that the hydroxyl group of RD2 was modified to the methoxy group of RD3 and at C-3, the methoxy group of RD2 was changed to the hydroxyl group of RD3. RD2 significantly reduced the viability of NSCLC (A549, H23, and H460) cells with half maximal inhibitory concentrations (IC_50_) at 108.6 ± 10.82, 103.5 ± 6.08, and 138.3 ± 25.63 µM, respectively (Figure 2a–d), while RD3 reduced cell viability in NSCLC cells (A549, H23, andH460) with the IC_50_ as more than 200 µM. Furthermore, resveratrol decreased cell viability in NSCLC cells (A549, H23, and H460) with the IC_50_ at more than 200 µM which was the same RD3 (Figure 2a–d). This different action on cancer cells as a result of chemical structure modification has provided an insight for the structure–activity relationship that may facilitate the further development of the potent compound from the lead structure of resveratrol derivatives. 

To confirm the selective cytotoxicity of resveratrol derivatives (RD2 and RD3) in cancer cells, resveratrol derivatives (RD2 and RD3) and resveratrol were determined for the cytotoxic profile in normal human cells, dermal papilla (DP). It was found that resveratrol derivatives (RD2 and RD3) and resveratrol did not significantly affect the cell viability percentages in DP cells, as shown in Figure 2e,f.

**Figure 3:**
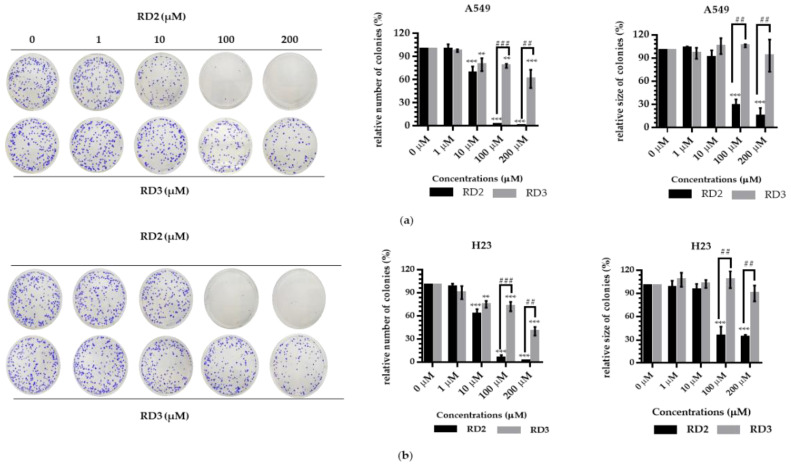
Resveratrol derivatives inhibit colony formation activity and induce apoptosis in NSCLC cells (A549 and H23). (**a**,**b**) Cells were treated with RD2 and RD3 (0–200 µM for 24 h). After 1 week, colonies were stained with 1.25% crystal violet and quantified by extraction with 10% acetic acid and the analysis are determined by number and size of colony cancer cell. Data are represented as the mean ± SEM (*n* = 3). * *p* < 0.05, ** *p* < 0.01, *** *p* < 0.001 when compared with the control group. # *p* < 0.05, ## *p* < 0.01, ### *p* < 0.001 when compared between RD2 and RD3.

Colony formation assay was used to confirm the anti-cancer action of resveratrol derivatives, as it is a cell proliferation assay which can detect the ability of a single cell to grow into a colony. NSCLC cells were seeded and treated with RD2 and RD3 (1–200 μM) for 24 h. The treated cells were subjected to colony formation assay for 7 days. The crystal violet-staining colony represented the colony suppression effect of RD2 and RD3 compared to the untreated control (Figure 3a,b). The RD2 compound dramatically abolished the forming colony. Colony number was absent in response to RD2 at the concentrations of 100 and 200 μM, whereas there was no significant response to RD3 (Figure 3a,b).

RD2 (10–200 μM) significantly inhibited the number of colony growth compared with the control cells in a dose-dependent manner. The results showed that the number of the forming colony was significantly reduced by 31.04%, 98.18%, and 99.40% in response to the treatment of 10, 100, and 200 μM, respectively, in A549 cells and 25.43%, 94.16%, and 98.23% in response to the treatment of 10, 100, and 200 μM, respectively, in H23 cells. Moreover, RD2 (100–200 μM) significantly inhibited the colony size compared with the control cells. The results showed that the colony size was significantly decreased by 71.66% and 85.42% in response to the treatment of 100 and 200 μM, respectively, in A549 cells and 64.42% and 65.98% in response to the treatment of 100 and 200 μM, respectively, in H23 cells (Figure 3a,b).

RD3 (10–200 μM) significantly inhibited the number of colony growth compared with the control cells, in a dose-dependent manner. The results show that the number of the forming colony was significantly reduced by 20.44%, 21.94%, and 38.91% in response to the treatment of 10, 100, and 200 μM, respectively, in A549 cells and 24.83%, 27.70% and 59.74% in response to the treatment of 10, 100, and 200 μM, respectively, in H23 cells. However, for RD3, there was no significant effect in the changing colony size in both A549 and H23 cells (Figure 3a,b).

In summary, these results suggest the promising anti-cancer effects of RD2 and RD3, a new resveratrol derivatives.

### 2.3. Apoptosis Induction and Apoptotic-Related Protein Alteration in Response to RD2 and RD3 Treatments

**Figure 4:**
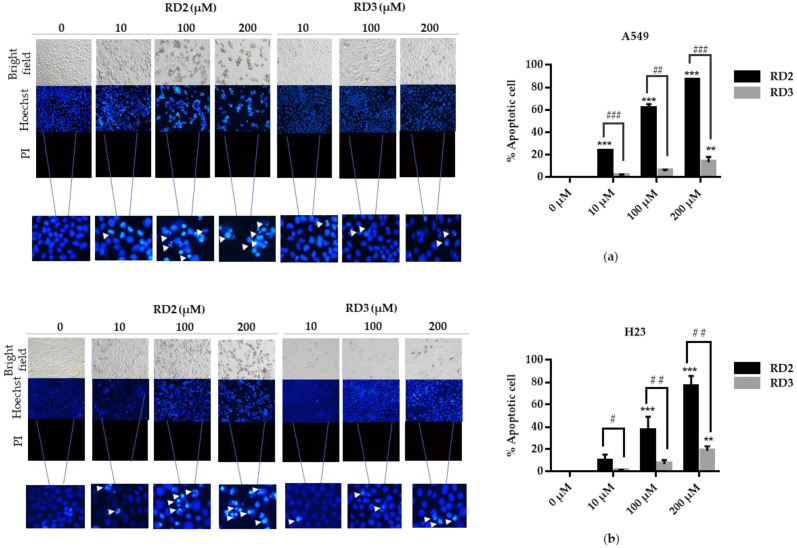
Resveratrol derivatives induces apoptosis in NSCLC. (**a**,**b**) Cells were treated with RD2 and RD3 (0–200 mM) for 24 h and then stained with Hoechst 33342 and PI. The images were visualized using an inverted fluorescence microscope. The condensed blue fluorescence of Hoechst 33342 represents the fragmented chromatin in apoptotic cells as a percentage compared with untreated control cells and another group. Data are represented as the mean ± SEM (*n* = 3). * *p* < 0.05, ** *p* < 0.01, *** *p* < 0.001 when compared with the control group. # *p* < 0.05, ## *p* < 0.01, ### *p* < 0.001 when compared between RD2 and RD3.

Apoptosis is considered as an important mechanism of action of anti-cancer drugs [16]. The important characteristics of apoptotic cells include the nuclear morphological changes, chromatin condensation, and DNA fragmentation. Hoechst 33342 and propidium iodide (PI) double staining was utilized to determine the mode of cell death by monitoring the nucleus morphology of apoptotic cells as well as their membrane integrity. Cells were seeded in a 96-well plate at the density of 1 × 10^4^ cells/well and treated with various concentrations of RD2 and RD3 (10–200 μM) for 24 h. The cells were co-stained with Hoechst 33342 and PI. Bright blue fluorescence of Hoechst 33342 stained cells was observed and the apoptotic cells exhibiting condensed and fragmented chromatin were monitored. The red fluorescence of PI indicated a late apoptosis cell and necrotic cells. Figure 4a,b show that RD2 and RD3 significantly caused apoptosis indicated by the increase in nucleus chromatin condensed cells and apoptotic nuclei. 

RD2 significantly increased the apoptotic rates compared with the non-treated control cells, in a dose-dependent manner. The apoptotic ratios were 23.25%, 61.26%, and 86.30% in the treatment of 10, 100, and 200 μM, respectively, in A549 cells. In addition, RD2 significantly also increased the apoptotic rates compared with the control cells in a dose-dependent manner. The apoptotic ratios were 36.63% and 75.79% in the treatment of 100 and 200 μM, respectively, in H23 cells (Figure 3c,d). RD3 only significantly increased apoptotic ratios by 13.25% at 200 μM in A549 cells and 18.57% at 200 μM in (Figure 4a,b). According to the previous experiment showing that RD2 had a potent cytotoxicity effect, RD2 had also caused more dead cells than RD3 at the same concentrations (10–200 μM); this was observed from the blue fluorescence of condensed nuclei indicating the early stage of cell death. 

### 2.4. RD2 Inhibits Akt/mTOR Singnaling Pathways 

Having shown that the resveratrol derivatives decrease cell viability, induce apoptosis, and inhibit the growth of the cells, we next investigated the potential mechanistic effect of the compounds by focusing on the proliferative and survival signal Akt/mTOR. 

**Figure 5:**
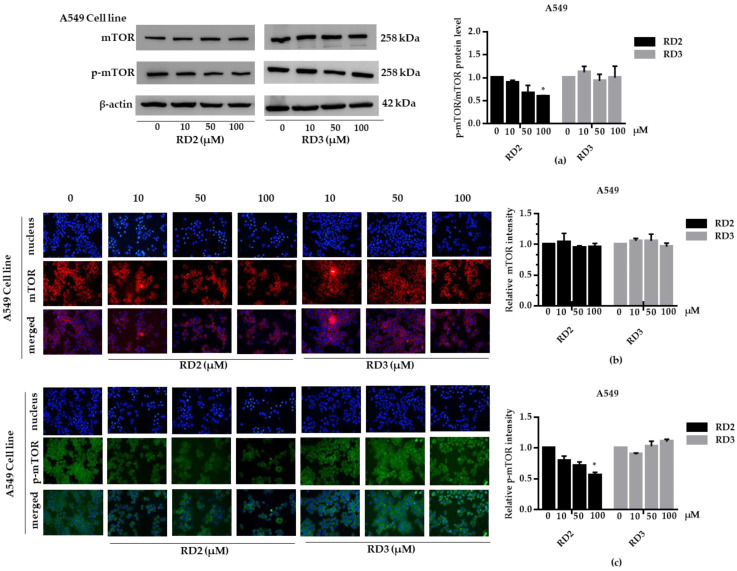
RD2 downregulates *p*-mTOR in NSCLC cells (A549). The cells were treated with RD2 and RD3 (0–100 μM) for 24 h. (**a**) Western blot analysis was performed to measure the *p*-mTOR/mTOR-related proteins. β-Actin protein was evaluated to confirm the equal loading of each protein sample. Densitometry of each protein level was calculated and the results are presented as a relative protein level. (**b**) A549 cells were stained with mTOR (red fluorescence) and Hoechst 33342 (blue fluorescence). The expression of mTOR was determined by immunofluorescence. (**c**) A549 cells were stained with *p*-mTOR (green fluorescence) and Hoechst 33342 (blue fluorescence). The expression of *p*-mTOR was examined using immunofluorescence. Relative protein levels were quantified by densitometry. Data are represented as the mean ± SEM. * *p* < 0.05, when compared with the control group.

**Figure 6:**
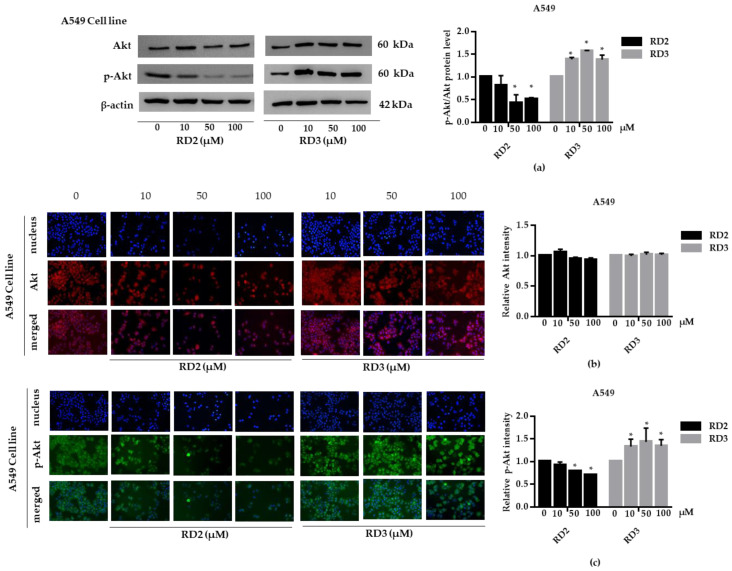
RD2 downregulates *p*-Akt in NSCLC cells (A549). The cells were treated with RD2 and RD3 (0–100 μM) for 24 h. (**a**) Western blot analysis was performed to measure the *p*-Akt/Akt-related proteins. β-Actin protein was evaluated to confirm the equal loading of each protein sample. Densitometry of each protein level was calculated and the results are presented as a relative protein level. (**b**) A549 cells were stained with Akt (red fluorescence) and Hoechst 33342 (blue fluorescence). The expression of Akt was determined by immunofluorescence. (**c**) A549 cells were stained with *p*-Akt (green fluorescence) and Hoechst 33342 (blue fluorescence). The expression of *p*-Akt was examined using immunofluorescence. Relative protein levels were quantified by densitometry. Data are represented as the mean ± SEM. * *p* < 0.05, when compared with the control group.

**Figure 7:**
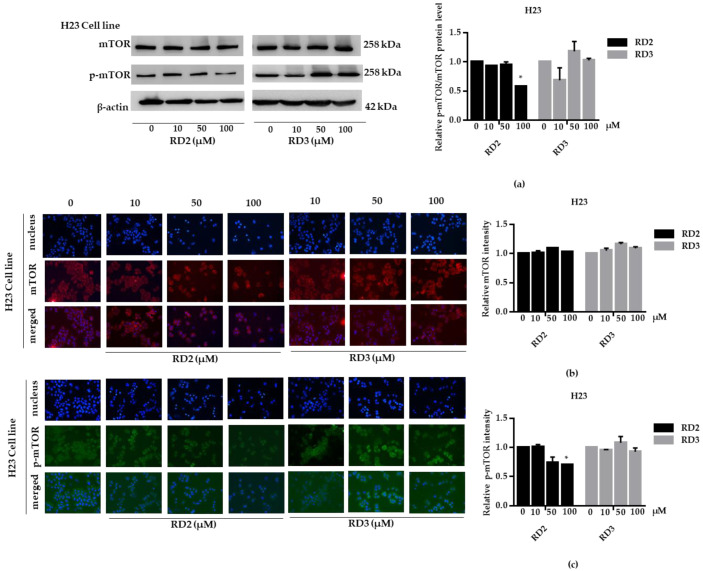
RD2 downregulates *p*-mTOR in NSCLC cells (H23). The cells were treated with RD2 and RD3 (0–100 μM) for 24 h. (**a**) Western blot analysis was performed to measure the *p*-mTOR/mTOR-related proteins. β-Actin protein was evaluated to confirm the equal loading of each protein sample. Densitometry of each protein level was calculated and the results are presented as a relative protein level. (**b**) H23 cells were stained with mTOR (red fluorescence) and Hoechst 33342 (blue fluorescence). The expression of mTOR was determined by immunofluorescence. (**c**) H23 cells were stained with *p*-mTOR (green fluorescence) and Hoechst 33342 (blue fluorescence). The expression of *p*-mTOR was examined using immunofluorescence. Relative protein levels were quantified by densitometry. Data are represented as the mean ± SEM. * *p* < 0.05, when compared with the control group.

**Figure 8:**
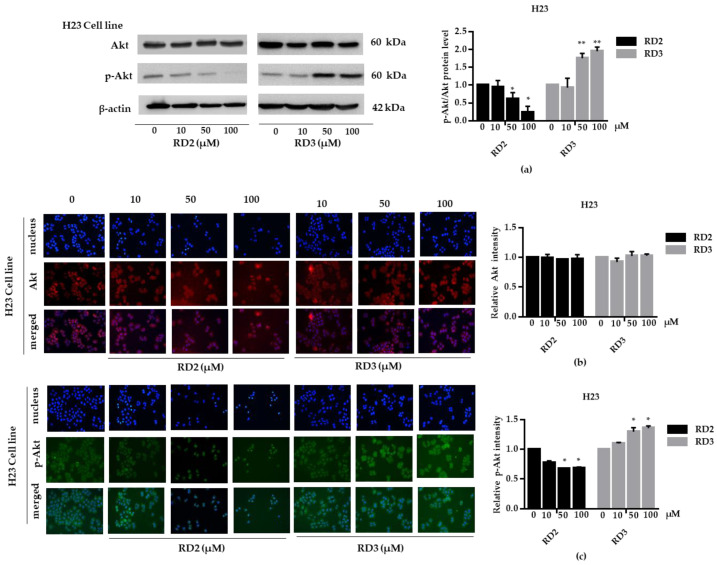
RD2 downregulates *p*-Akt in NSCLC cells (H23). The cells were treated with RD2 and RD3 (0–100 μM) for 24 h. (**a**) Western blot analysis was performed to measure the *p*-Akt/Akt-related proteins. β-Actin protein was evaluated to confirm the equal loading of each protein sample. Densitometry of each protein level was calculated and the results are presented as a relative protein level. (**b**) A549 cells were stained with Akt (red fluorescence) and Hoechst 33342 (blue fluorescence). The expression of Akt was determined by immunofluorescence. (**c**) H23 cells were stained with *p*-Akt (green fluorescence) and Hoechst 33342 (blue fluorescence). The expression of *p*-Akt was examined using immunofluorescence. Relative protein levels were quantified by densitometry. Data are represented as the mean ± SEM. * *p* < 0.05, ** *p* < 0.01, when compared with the control group.

NSCLC cells (A549 and H23) were treated with RD2 and RD3 (10–100 μM) for 12 h. After which, all cells were collected and the expression levels of Akt, *p*-Akt, mTOR, and *p*-mTOR were investigated by Western blot analysis (Figure 5a, Figure 6a, Figure 7a and Figure 8a). The results reveal a significant decrease of *p*-mTOR/mTOR protein expression levels at 100 μM and a significant decrease of *p*-Akt/Akt protein expression levels at 50 and 100 μM of RD2 treatment in NSCLC cells (A549 and H23) compared with the untreated control. Interestingly, RD3 increased *p*-Akt/Akt protein expression levels at 10–200 μM in A549 cell and at 100–100 μM in H23 cells. Interestingly, RD3 had no effect on *p*-mTOR/mTOR protein expression levels. These results suggest that the anticancer activity of RD2 might act, at least in part, via Akt/mTOR inhibition. Immunofluorescence staining of Akt, *p*-Akt, mTOR, and *p*-mTOR was performed in RD2 and RD3 treated cells. The results further confirm the effect of RD2 and RD3 on the Akt/mTOR pathway. While Akt and mTOR fluorescence intensity were not notably changed in RD2 and RD3 in NSCLC (A549 and H23), RD2 induced a dramatic decrease of the *p*-mTOR signal in NSCLC cells (A549 and H23) at 50–100 μM, while RD3 only minimally affected the intensity of *p*-mTOR signal (Figure 5c, Figure 6c, Figure 7c and Figure 8c). RD3 treatment significantly increased *p*-Akt intensity at 10–100 µM in A549 cells (Figure 6b) and at 100–100 µM in H23 cells (Figure 8b) compared with untreated control cells. 

**Figure 9:**
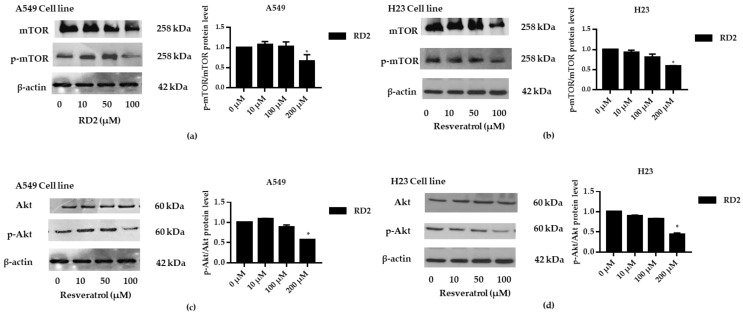
Resveratrol downregulates *p*-Akt and *p*-mTOR in NSCLC cells (A549 and H23). The cells were treated with resveratrol (0–100 μM) for 24 h. (**a**–**d**) Western blot analysis was performed to measure the *p*-Akt/Akt-related proteins. β-Actin protein was evaluated to confirm the equal loading of each protein sample. Densitometry of each protein level was calculated and the results are presented as a relative protein level. Relative protein levels were quantified by densitometry. Data are represented as the mean ± SEM. * *p* < 0.05, when compared with the control group.

To investigate whether resveratrol inhibits the Akt/mTOR signaling pathway, NSCLC cells (A549 and H23) were treated with resveratrol (10–100 μM) for 12 h. After which, all cells were collected, and the expression levels of Akt, *p*-Akt, mTOR, and *p*-mTOR were investigated by Western blot analysis. The results reveal a significant decrease of *p*-mTOR/mTOR protein expression levels at 100 μM and a significant decrease of *p*-Akt/Akt protein expression levels at 100 μM of resveratrol treatment compared with the untreated control (Figure 9a–d), while RD2 could reduce *p*-Akt/Akt protein expression levels at a lower concentration (50–100 μM). 

### 2.5. Molecular Docking Simulations Reveals the Reveratrol Derivatives Interactions with Akt-1 Protein

We further investigated whether Akt inhibition of RD2 might be a result of direct interaction between RD2 and Akt protein. To determine the plausible modes of Akt-1 interaction, molecular docking analyses were performed using co-crystal structures of ATP-competitive inhibitor (PDB ID 3CQW) and an allosteric inhibitor (PDB ID 5KCV). 

**Figure 10:**
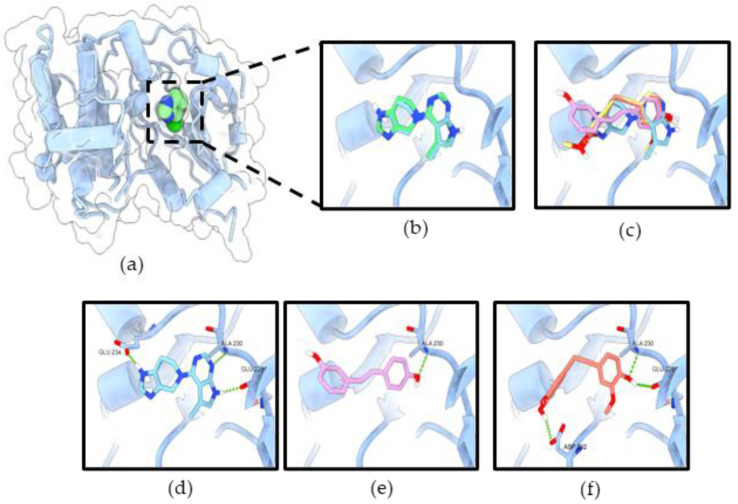
RD2 inhibits Akt activity via interaction with the ATP-binding site of Akt-1. (**a**) Co-crystal structure of CQW (ATP-competitive inhibitor) in Akt-1 (PDB 3CQW); (**b**) the original ligand (green) and redocking (blue); (**c**) superimposition of CQW, resveratrol (pink), and RD2 (red); the binding mode of compounds CQW (**d**), resveratrol (**e**), and RD2 (**f**) in the ATP-binding site of Akt-1. The green dashed lines indicate hydrogen bonds.

**Figure 11:**
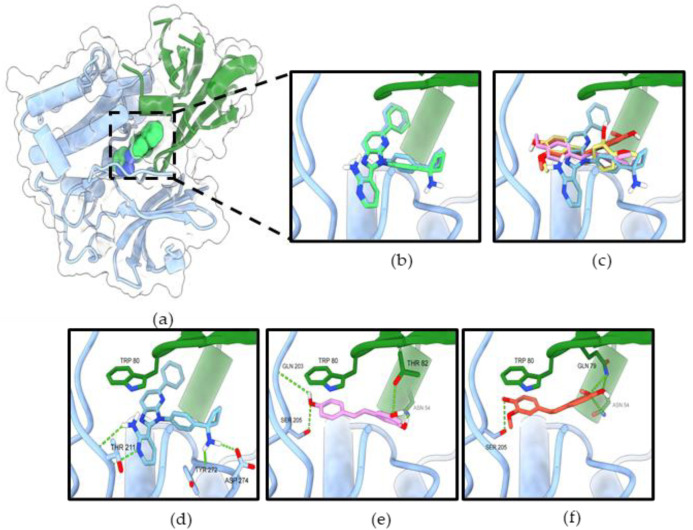
RD2 inhibits Akt activity via interaction with the allosteric binding site of Akt-1 (**a**) Co-crystal structure of miransertib (allosteric inhibitor) in Akt-1 (PDB 5KCV). (**b**) The original ligand (green) and redocking (blue). (**c**) Superimposition of miransertib, resveratrol (pink), and RD2 (red). The binding mode of compounds miransertib (**d**), resveratrol (**e**), and RD2 (**f**) in the allosteric binding site of Akt-1. The PH domain is shown in green, the kinase domain in blue. The green dashed lines indicate hydrogen bonds.

The initial validation of the docking protocol was performed by redocking. The redocking result showed that co-crystal ligands (reference compounds) exhibited similar poses to the original crystal structures (Figure 10b and Figure 11b), which was further confirmed by the root mean square deviation (RMSD) value of 0.451 Å and 0.957 Å, respectively.

In the ATP-binding site of Akt-1, the binding affinity for resveratrol and RD2 were −8.054 and −8.041 kcal/mol, respectively (Table 1). The binding modes of all ligands revealed that they occupied the same binding site of the co-crystal ligand inhibitor (Figure 10c). The results reveal that all ligands formed a hydrogen bond with key residue Ala230, which is a similar binding pattern to the reference compound (Figure 10d–f). Resveratrol formed only one hydrogen bond interaction with Ala230 in the hinge region of the ATP-binding site. Moreover, previous studies have reported that resveratrol has inhibited Akt activity via interaction with the ATP-binding site of Akt-1 [11,17]. RD2 formed three hydrogen bonds with Glu228, Ala230 in the hinge region, and Asp292 in the DFG motif. 

In the allosteric binding site of Akt-1, the binding affinity for resveratrol and RD2 were −8.446, and −8.546 (Table 2). The allosteric binding site of Akt-1 mainly interacts with Trp80, which plays an essential role in the binding affinity. Furthermore, three ligands were formed by hydrophobic interaction with key residue Trp80 of the PH domain. All ligands adopted a similar binding mode of miransertib (reference compound) with an interface between the PH and the kinase domain (Figure 11c–f). Resveratrol formed four hydrogen bonds with Asn54 and Thr82 in the PH domain and Gln203 and Ser205 in the kinase domain. Likewise, a previous study reported that resveratrol could bind to Akt-1 with an allosteric site [18]. RD2 formed four hydrogen bonds with Asn54, Gln79 (2 bonds) in the PH domain, and Ser205 in the kinase domain. These results illustrate that the Akt signaling pathway is involved in the inhibitory effect of resveratrol and RD2.

To test the potential binding of RD2 and RD3 on the activation site of Akt, we docked RD2, RD3, and Akt activator SC79 as ligands into the PH domain of Akt-1 (PDB ID 1UNQ) using AutoDock Vina. A previous study reported that SC79 directly binds to the PH domain and enhances Akt phosphorylation and activation [19].

**Figure 12:**
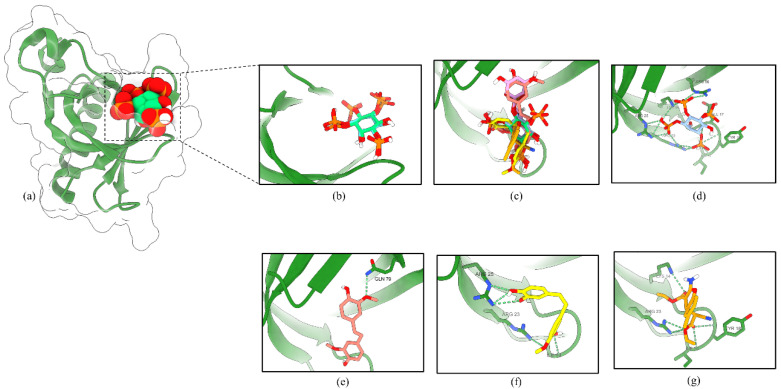
RD3 activates Akt via interaction with the PH domain of Akt-1. (**a**) Co-crystal structure of inositol (1,3,4,5)-tetrakisphosphate (IP4) in Akt-1 (PDB 1UNQ). (**b**) The original ligand (green) and redocking (blue). (**c**) Superimposition of IP4, RD2 (red), RD3 (yellow), and SC79 (orange). The binding mode of compounds IP4 (**d**), RD2 (**e**), RD3 (**f**), and SC79 (**g**) in the PH domain of Akt-1. The green dashed lines indicate hydrogen bonds.

In the PH domain of Akt-1, the binding affinity for RD2, RD3, and SC79 were −5.297, −6.072, and −5.62 kcal/mol, respectively (Table 3). The hydrogen-bond interactions of the PH domain, Lys14, Arg25, and Arg86, were documented to be essential for Akt activation [20]. As shown in Figure 12, RD3 formed hydrogen bonds with Ile19, Arg23, and Arg25, and the activator SC79 formed hydrogen bonds with the Lys14, Tyr18, Ile19, and Arg23 side-chain residues. The molecular docking result clarified the mechanism of Akt activation by RD3, which might present a reasonable interaction pose in the PH domain of Akt-1, similarly to Akt activator SC79 and native ligand IP4.

## 3. Discussion

Lung cancer is one of the most important human cancers and the disease causes high mortality with poor overall prognosis. Conventional drugs are frequently not effective at an advanced stage as well as for metastatic lung cancers [21]. New treatment options including targeted therapy are found to potentially improve survival compared to those treated without targeted therapies [2]. It was found that plant-derived bioactive compounds and their synthetic analogs show promising antitumor activity as a targeted therapeutic drug [22]. One such bioactive agent is resveratrol which is a polyphenol exhibiting health beneficial effects including antioxidant, anti-inflammation, and antitumor effects. In addition, in lung cancer cells, resveratrol shows anti-cancer activities. Resveratrol was demonstrated to inhibit cancer cell growth, mediate cell cycle arrest, and induce apoptosis [23]. For lung cancer, it was demonstrated that resveratrol caused the induction of p53-dependent apoptosis in A549 cells [24]

Resveratrol has been recognized as an interesting lead compound for chemical structure modifications to gain more potency and altered water solubility [25]. In addition, the anti-cancer activity of the derivatives as well as the presence of each modified moiety may lead to new knowledge of the structure–activity relationship that is useful for further development of the compound. Therefore, we modified the molecular structure of resveratrol into five derivatives (RD1-RD5) (Figure 1a). Among these compounds, RD2 was shown to have the most potent cytotoxic effect on NSCLC cells (Figure 2a–d). It is interesting to us that the RD2 and RD3 had a very similar structure. however, RD2 had more anti-cancer effect than that of RD3. RD2 exerted more effects on cancer cell viability (Figure 2d), on proliferating tumor cells (Figure 3a,b), and apoptotic cell death (Figure 4a,b). The anti-proliferation effect of resveratrol derivatives was consistent with the previous study. Regarding their parental compound, resveratrol was previously reported to inhibit cell proliferation in a concentration- and time-dependent manner in lung cancer A549 cells via the induction of cell cycle arrest at the G0/G1 phase [26]. Moreover, pterostilbene, an analog of resveratrol, was shown to significantly decrease cell viability and induced S phase arrest and the activation of caspase −3, −8, and −9 in human lung squamous cell carcinoma (H520) cells [27].

For cancer aggressiveness and treatment failure, it is known that the cellular survival signals can potentiate cell growth, immune escape, and chemotherapeutic drug resistance. The phosphoinositide 3-kinase (PI3K)/Akt signaling pathway is an important survival and proliferating signaling pathway that was shown to be up-regulated and overactivated in various types of cancer. The PI3K/Akt signal controls the hallmarks of cancer, including cell survival, proliferation, motility and metastasis, angiogenesis, and inflammatory factor recruitment [28]. Deregulation of the PI3K/Akt/mTOR pathway was shown to be critical for lung tumorigenesis and advanced disease [29]. Taken together, the inhibition of the PI3K/Akt/mTOR pathway may offer a possible option for the improvement of lung cancer therapy. In the present study, it was revealed that RD2 significantly decreased the level of active Akt (phosphorylated Akt) and active mTOR (phosphorylated mTOR) in A549 and H23 cells. The inhibition of Akt/mTOR signaling was consistent with the inhibitory effects of the compound on cell survival and the expression of related proteins (Figure 5a, Figure 6a, Figure 7a and Figure 8a). Consistently, several studies reported that resveratrol has an anti-tumor effect via Akt and mTOR. Resveratrol downregulated the PI3K/Akt/mTOR signaling pathway, which resulted in apoptosis in glioma cells [9]. Resveratrol also inhibited proliferation and migration through sirtuin 1-mediated post-translational modification of the PI3K/Akt pathway in hepatocellular carcinoma cells [30]. Treatment with resveratrol alone or in combination with rapamycin efficiently inhibited cell growth by inducing growth stop by targeting Akt activation and preventing mTORC1 signaling cascade phosphorylation-activation [31] Consistently, resveratrol was shown to decrease cellular levels of PI3K and *p*-Akt in NSCLC [12]. The combination of resveratrol and erlotinib could decrease the *p*-Akt, *p*-mTOR, and *p*-S6K relative to either agent alone, indicating the synergistic suppression of the Akt/mTOR pathway in NSCLC [32].

In this study, the binding affinity and the number of hydrogen bonds were suggested to play a role on the inhibition mechanism of Akt-1 by the RD2 compound. Docking analysis of RD2 with the ATP binding site and the halosteric site of Akt-1 shows the ability to interact with both binding sites; however, the Vina scoring function and hydrogen bonds of the two ligands complex with the allosteric binding site suggest them as the preferred ligand for an allosteric binding site than the ATP-binding site of Akt-1. Similarly, it has been reported that resveratrol has a dual action inhibitor of Akt. It is a potential ATP-competitive inhibitor and an allosteric inhibitor of Akt [33].

## 4. Materials and Methods

### 4.1. Cell Lines and Cultures 

NSCLCcells (H23, H460, and A549) and Human follicle dermal papilla fibroblast cells (DP cells)were obtained from the American Type Culture Collection (Manassas, VA, USA) andPromoCell (PromoCell, Heidelberg, Germany). A549 and DPcells were grown in Dulbecco’s Modified Eagle’s Medium (DMEM) medium whereas, H23 and H460 cells were cultured in Roswell Park Memorial Institute (RPMI) 1640 medium in 5% CO_2_ incubator contain 37 °C. These mediums consist with 10% fetal bovine serum (FBS), 2 mM L-glutamine, and 100 units/mL each of penicillin and streptomycin.

### 4.2. Reagents and Antibodies

Roswell Park Memorial Institute (RPMI), Dulbecco’s Modified Eagle’s Medium (DMEM) medium, fetal bovine serum (FBS), penicillin/streptomycin, L-glutamine, phosphate-buffered saline (PBS), and trypsin-EDTA were obtained from Gibco (Grand Island, NY, USA). crystal violet solution (1% *w*/*v*), formaldehyde solution (37% *w*/*v*), and skim milk powder were purchased from Sigma Chemical, Inc. (St. Louis, MO, USA). 3-(4,5-dimethylthiazol-2-yl)-2,5-Diphenyltetrazoliumbromide (MTT), dimethyl sulfoxide (DMSO), Hoechst 33342, propidium iodide (PI), and bovine serum albumin (BSA) were obtained from Sigma-Aldrich, Co. (St. Louis, MO, USA). Primary antibodies specific to Akt (#9272), phosphorylated Akt (#4060), mTOR (#2983S), and *p*-mTOR (#5536S) were obtained from Cell Signaling Technology (Danvers, MA, USA). The respective secondary antibody (anti-rabbit IgG (#7074)) was obtained from Cell Signaling Technology (Danvers, MA, USA).

### 4.3. Resveratrol and Resveratrol Derivatives

Resveratrol standard was purchased from Sigma Aldrich (Steinheim, Germany). Resveratrol derivatives (RD1-RD5) were obtained starting from commercially available aromatic aldehydes (vanilline, isovanilline, and syringaldehyde) and the easily preparable known aldehyde 10 [34]. The hydroxy groups of vanilline, isovanilline, and syringaldehyde were protected as benzyl ethers 1, 4, and 9. Aldehydes were reduced to an alcohol 2, 5, and 7, which was then converted to a bromide followed by Michaelis–Arbuzov reaction [35] with triethyl phosphite to obtain the Horner–Wadsworth–Emmons reagent 3, 6, and 8. The obtained Horner–Wadsworth–Emmons reagents were reacted with the corresponding aldehydes to obtain stilbene derivatives 11–13 by Horner–Wadsworth–Emmons reaction [36]. Finally, each compound was catalytically hydrogenated to yield RD1–RD5 (Figure 1b).

#### 4.3.1. 4-(Benzyloxy)-3-methoxybenzaldehyde (**1**)

A solution vanilline (10.0 g, 65.7 mmol) in CH_3_CN (54 mL) was added to NaHCO_3_ (6.29 g, 74.9 mmol, 1.14 equiv.) and KI (1.09 g, 6.57 mmol, 0.1 equiv.), and the obtained solution was heated to 60 °C. After benzyl chloride (8.00 mL, 69.5 mmol, 1.06 equiv.) was added to this solution, it was refluxed for 5 h. After cooling to room temperature, the reaction mixture was evaporated under vacuum. The residue was diluted with HCl solution (2.1 mL, 1 mol/L) and extracted with EtOAc (50 mL × 3), washed with brine, dried over anhydrous Na_2_SO_4_, and concentrated. The crude product was purified over SiO_2_ column (n-Hex.: EtOAc = 7:3) to give 1 (8.33 g, 52%) as a colorless solid. ^1^H NMR (300 MHz, CDCl_3_) δ: 9.84 (1H, s), 7.30–7.45 (7H, m), 6.99 (1H, d, *J* = 8.2 Hz), 5.25 (2H, s), 3.95 (3H, s).

#### 4.3.2. (4-(Benzyloxy)-3-methoxyphenyl)methanol (**2**)

A solution of 1 (3.00 g, 12.4 mmol) in methanol (30 mL), tetrahydrofuran [(THF) 30 mL], and H_2_O (3 mL) was added to NaBH_4_ (515 mg, 13.6 mmol, 1.1 equiv.) at 0 °C, and the reaction mixture was stirred for 1 h. The reaction was diluted with Et_2_O (30 mL) and quenched with HCl solution (11 mL, 1 mol/L). The obtained solution was evaporated under vacuum. The residue was diluted with H_2_O (10 mL) and extracted with EtOAc (60 mL × 3), washed with brine, dried over anhydrous Na_2_SO_4_, and concentrated to give 2 (2.99 g, 100%) as a colorless solid. ^1^H NMR (400 MHz, CDCl_3_) δ: 7.43 (2H, d, *J* = 7.1 Hz), 7.36 (2H, t, *J* = 7.1 Hz), 7.30 (1H, t, *J* = 7.1 Hz), 6.95 (1H, d, *J* = 1.6 Hz), 6.86 (1H, d, *J* = 8.4 Hz), 6.82 (1H, dd, *J* = 1.6, 8.4 Hz), 5.16 (2H, s), 4.61 (2H, s), 3.91 (3H, s).

#### 4.3.3. Diethyl (4-(Benzyloxy)-3-methoxybenzyl)phosphonate (**3**)

A solution of NBS (7.65 g, 43.0 mmol, 3.5 equiv.) in CH_2_Cl_2_ (44 mL) was added to dimethylsulfide (3.77 mL, 51.6 mmol, 4.2 equiv.) at 0 °C over 7 min. The reaction mixture was stirred at this temperature for 10 min. A solution of 2 (3.00 g, 12.3 mmol) in CH_2_Cl_2_ (44 mL) was cooled at −18 °C and was added to the above solution. The reaction mixture was stirred at −18 °C for 3 h. The reaction mixture was warmed to 0 °C and diluted with H_2_O and extracted with CH_2_Cl_2_ (80 mL × 3), washed with saturated NaHCO_3_ solution and H_2_O, dried over anhydrous Na_2_SO_4_, and concentrated. The crude product was dissolved in triethyl phosphite (2.79 mL, 16.1 mmol, 1.24 equiv.). The reaction mixture was stirred at 140 °C for 4 h. After cooling to room temperature, the reaction mixture was evaporated under vacuum. The residue was purified over SiO_2_ column (n-Hex.: EtOAc = 1:9) to give 3 (2.01 g, 45%) as a yellow oil. ^1^H NMR (400 MHz, CDCl_3_) δ: 7.29–7.44 (5H, m), 6.89 (1H, s), 6.82 (1H, d, *J* = 8.0 Hz), 6.75 (1H, d, *J* = 8.0 Hz), 5.13 (2H, s), 3.93–4.11 (4H, m), 3.89 (3H, s), 3.04 (2H, d, *J* = 21.2 Hz), 1.20 (6H, t, *J* = 7.1 Hz). 

#### 4.3.4. 3-(Benzyloxy)-4-methoxybenzaldehyde (**4**)

A solution of isovanilline (10.0 g, 65.7 mmol) in acetone (100 mL) was added to K_2_CO_3_ (2.7 g, 164 mmol, 2.5 equiv.) and benzyl bromide (8.59 mL, 72.3 mmol, 1.1 equiv.), and the obtained solution was refluxed for 20 h. After cooling to room temperature, the reaction mixture was filtered, and the obtained filtrate was evaporated under vacuum. The residue was diluted with H_2_O and extracted with EtOAc (50 mL × 3), washed with brine, dried over anhydrous Na_2_SO_4_, and concentrated. The crude product was purified over SiO_2_ column (n-Hex.: EtOAc = 7:3) to give 4 (8.43 g, 53%) as a yellow solid. ^1^H NMR (300 MHz, CDCl_3_) δ: 9.82 (1H, s), 7.30–7.49 (7H, m), 7.00 (1H, d, *J* = 8.4 Hz), 5.20 (2H, s), 3.97 (3H, s).

#### 4.3.5. (3-(Benzyloxy)-4-methoxyphenyl)methanol (**5**)

A solution of 4 (3.00 g, 12.4 mmol) in methanol (30 mL), tetrahydrofuran [(THF) 30 mL], and H_2_O (3 mL) was added to NaBH_4_ (515 mg, 13.6 mmol, 1.1 equiv.) at 0 °C, and the reaction mixture was stirred for 1 h. The reaction was diluted with Et2O (30 mL) and quenched with HCl solution (11 mL, 1 mol/L). The obtained solution was evaporated under vacuum. The residue was diluted with H_2_O (10 mL) and extracted with EtOAc (60 mL × 3), washed with brine, dried over anhydrous Na_2_SO_4_, and concentrated to give 5 (2.76 g, 92%) as a yellow solid. ^1^H NMR (300 MHz, CDCl_3_) δ: 7.43 (2H, d, *J* = 7.1 Hz), 7.36 (2H, t, *J* = 7.1 Hz), 7.30 (1H, t, *J* = 7.1 Hz), 6.86–6.95 (3H, m), 5.16 (2H, s), 4.57 (2H, s), 3.88 (3H, s).

#### 4.3.6. Diethyl (3-(Benzyloxy)-4-methoxybenzyl)phosphonate (**6**)

A solution of NBS (6.37 g, 35.8 mmol, 3.5 equiv.) in CH_2_Cl_2_ (36.5 mL) was added to dimethylsulfide (3.14 mL, 43.0 mmol, 4.2 equiv.) at 0 °C over 6 min. The reaction mixture was stirred at this temperature for 10 min. A solution of 5 (2.50 g, 10.2 mmol) in CH_2_Cl_2_ (36.5 mL) was cooled at −18 °C and was added to the above solution. The reaction mixture was stirred at −18 °C for 3 h. The reaction mixture was warmed to 0 °C and diluted with H_2_O and extracted with CH_2_Cl_2_ (50 mL × 3), washed with saturated NaHCO_3_ solution and H_2_O, dried over anhydrous Na_2_SO_4_, and concentrated. The crude product was dissolved in triethyl phosphite (2.20 mL, 12.7 mmol, 1.24 equiv.). The reaction mixture was stirred at 140 °C for 4 h. After cooling to room temperature, the reaction mixture was evaporated under vacuum. The residue was purified over SiO_2_ column (n-Hex.: EtOAc = 1:9) to give 6 (1.17 g, 31%) as a colorless oil. ^1^H NMR (300 MHz, CDCl_3_) δ: 7.28–7.46 (5H, m), 6.89 (1H, s), 6.84 (2H, s), 5.15 (2H, s), 3.89–4.01 (4H, m), 3.87 (3H, s), 3.04 (2H, d, *J* = 21.3 Hz), 1.21 (6H, t, *J* = 7.1 Hz).

#### 4.3.7. 4-(Hydroxymethyl)-2,6-dimethoxyphenol (**7**)

A solution of syringaldehyde (5.00 g, 32.9 mmol) in methanol (78.4 mL), tetrahydrofuran [(THF) 78.4 mL], and H_2_O (7.84 mL) was added to NaBH_4_ (1.37 g, 36.1 mmol, 1.1 equiv.) at 0 °C, and the reaction mixture was stirred for 1.5 h. The reaction was diluted with Et_2_O (30 mL) and quenched with HCl solution (20 mL, 1 mol/L). The obtained solution was evaporated under vacuum. The residue was diluted with H_2_O (30 mL) and extracted with EtOAc (150 mL × 3), washed with brine, dried over anhydrous Na_2_SO_4_, and concentrated to give 7 (4.09 g, 81%) as a colorless solid. ^1^H NMR (300 MHz, CDCl_3_) δ: 6.61 (2H, s), 5.50 (1H, brs), 4.62 (2H, s), 3.90 (6H, s). 

#### 4.3.8. Diethyl (4-Hydroxy-3,5-dimethoxybenzyl)phosphonate (**8**)

A solution of 7 (4.00 g, 21.7 mmol) in Et_2_O (151 mL) was added to PBr_3_ (2.70 mL, 28.2 mmol, 1.3 equiv.) at 0 °C. The reaction mixture was stirred at this temperature for 1.5 h. The reaction mixture was concentrated in vacuo and the obtained mixture was diluted with H_2_O (50 mL) and extracted with EtOAc (150 mL × 3), washed with saturated NaCl solution and H_2_O, dried over anhydrous Na_2_SO_4_, and concentrated. The crude product was dissolved in triethyl phosphite (4.70 mL, 26.9 mmol, 1.24 equiv.). The reaction mixture was stirred at 140 °C for 4 h. After cooling to room temperature, the reaction mixture was evaporated under vacuum. The residue was purified over SiO_2_ column (n-Hex.: EtOAc = 1:9) to give 8 (1.60 g, 25%) as a yellow oil. ^1^H NMR (300 MHz, CDCl_3_) δ: 6.54 (2H, d, *J* = 2.6 Hz), 3.95–4.08 (4H, m), 3.88 (6H, s), 3.08 (2H, d, *J* = 21.2 Hz), 1.26 (6H, t, *J* = 7.0 Hz).

#### 4.3.9. 4-(Benzyloxy)-3,5-dimethoxybenzaldehyde (**9**)

A solution syringaldehyde (10.0 g, 54.9 mmol) in methanol (33.4 mL) was added to K_2_CO_3_ (9.10 g, 65.9 mmol, 1.2 equiv.) and benzyl bromide (7.82 mL, 65.9 mmol, 1.2 equiv.), and the obtained solution was refluxed for 20 h. After cooling to room temperature, the reaction mixture was filtered, and the obtained filtrate was evaporated under vacuum. The residue was dissolved with CHCl_3_ (70 mL) and washed with H_2_O (25 mL × 2), washed with brine, dried over anhydrous Na_2_SO_4_, and concentrated. The crude product was purified over SiO_2_ column (n-Hex.: EtOAc = 7:3) to give 9 (9.40 g, 63%) as a yellow oil. ^1^H NMR (400 MHz, CDCl_3_) δ: 9.86 (1H, s), 7.47 (2H, dd, *J* = 1.6, 7.3 Hz), 7.28–7.38 (3H, m), 7.11 (2H, s), 5.13 (2H, s), 3.90 (6H, s).

#### 4.3.10. (E)-4-(3-(Benzyloxy)-4-methoxy-5-methylstyryl)-2,6-dimethoxyphenol (**11**)

A solution of 8 (330 mg, 1.09 mmol, 1.2 equiv.) in THF (5.5 mL) was stirred at −78 °C and added to the *t*-BuOK solution in THF (3.26 mL, 3.26 mmol, 3.6 equiv., 1.0 M) over 30 min. The reaction mixture was stirred for 20 min at the same temperature, and was added 10 (232 mg, 0.904 mmol) in THF (1.0 mL) over 20 min and the mixture was stirred for 1 h at −78 °C and for 10 min at 0 °C. Then, the reaction mixture was stirred for 1.5 h at room temperature. The reaction mixture was cooled to 0 °C and diluted with saturated NH_4_Cl solution and extracted with EtOAc (60 mL × 3), washed with saturated NH_4_Cl solution and H_2_O, dried over anhydrous Na_2_SO_4_, and concentrated. The crude product was purified over SiO_2_ column (CH_2_Cl_2_) to give 12 (173 mg, 47%) as a colorless solid. ^1^H NMR (400 MHz, CDCl_3_ δ: 7.48 (2H, d, *J* = 7.3 Hz), 7.40 (2H, t, *J* = 7.3 Hz), 7.33 (1H, t, *J* = 7.3 Hz), 6.96 (2H, d, *J* = 2.9 Hz), 6.87 (1H, d, *J* = 16.1 Hz), 6.86 (1H, d, *J* = 16.1 Hz), 6.73 (2H, s), 5.15 (2H, s), 3.94 (6H, s), 3.86 (3H, s), 2.30 (3H, s). ^13^C NMR (100 MHz, CDCl3) δ: 151.9, 147.5, 147.2, 137.2, 134.6, 133.0, 132.1, 129.0, 128.5, 127.9, 127.9, 127.3, 126.6, 121.6, 109.8, 103.2, 70.8, 60.3, 56.3, 16.0. IR (KBr cm^−1^): 3528, 3019, 2399, 1609, 1516, 1464, 1428, 1342, 1215, 1157, 1116, 1006, 957, 929, 770, 668, 624, 532, 478, 444, 426, 419, 406. EI-MS *m/z* (%): 407 (25), 406 (100), 287 (23), 251 (13), 91 (20). HRMS (EI): Calcd for C_25_H_26_O_5_, 406.1780; Found: *m/z* 406.1778.

#### 4.3.11. (E)-1,2-Bis(4-(benzyloxy)-3-methoxyphenyl)ethene (**12**)

A solution of 3 (500 mg, 1.37 mmol, 1.2 equiv.) in THF (6.9 mL) was stirred at −78 °C and added to the *t*-BuOK solution in THF (1.8 mL, 1.8 mmol, 1.6 equiv., 1.0 M) over 30 min. The reaction mixture was stirred for 20 min. at the same temperature, and was added 1 (277 mg, 1.14 mmol) in THF (1.3 mL) over 20 min and the mixture was stirred for 1 h at −78 °C and for 10 min at 0 °C. Then, the reaction mixture was stirred for 1.5 h at room temperature. The reaction mixture was cooled to 0 °C and diluted with saturated NH_4_Cl solution and extracted with EtOAc (80 mL × 3), washed with saturated NH_4_Cl solution and H_2_O, dried over anhydrous Na_2_SO_4_, and concentrated. The crude product was purified over SiO_2_ column (CH_2_Cl_2_) to give 12 (470 mg, 91%) as a colorless solid. ^1^H NMR (400 MHz, CDCl_3_) δ: 7.44 (4H, d, *J* = 6.8 Hz), 7.37 (4H, t, *J* = 6.8 Hz), 7.30 (2H, t, *J* = 6.8 Hz), 7.06 (2H, d, *J* = 2.0 Hz), 6.96 (2H, dd, *J* = 2.0, 8.3 Hz), 6.89 (2H, s), 6.85 (2H, d, *J* = 8.3 Hz), 5.17 (4H, s), 3.94 (6H, s). ^13^C NMR (100 MHz, CDCl3) δ: 149.8, 147.8, 137.1, 131.2, 128.5, 127.8, 127.2, 126.8, 119.4, 114.0, 109.2, 71.0, 56.0. IR (KBr cm^−1^): 3684, 3019, 2400, 1512, 1215, 764, 668, 476, 457, 438, 432, 419, 410, 405. EI-MS *m*/*z* (%): 453 (12), 452 (M+, 40), 362 (25), 361(100), 91 (90). HRMS (EI): Calcd for C_30_H_28_O_4_, 452.1988; Found: *m/z* 452.1987.

#### 4.3.12. (E)-2-(Benzyloxy)-4-(4-(benzyloxy)-3-methoxystyryl)-1-methoxybenzene (**13**)

A solution of 3 (200 mg, 0.549 mmol, 1.2 equiv.) in THF (2.8 mL) was stirred at −78 °C and added to the *t*-BuOK solution in THF (732 µL, 0.732 mmol, 1.6 equiv., 1.0 M) over 30 min. The reaction mixture was stirred for 20 min at the same temperature, and was added to 4 (110 mg, 0.457 mmol) in THF (0.5 mL) over 20 min and the mixture was stirred for 1 h at −78 °C and for 10 min at 0 °C. Then, the reaction mixture was stirred for 16.5 h at room temperature. The reaction mixture was cooled to 0 °C and diluted with saturated NH_4_Cl solution and extracted with EtOAc (60 mL × 3), washed with saturated NH_4_Cl solution and H_2_O, dried over anhydrous Na_2_SO_4_, and concentrated. The crude product was purified over SiO_2_ column (CH_2_Cl_2_) to give 13 (122 mg, 59%) as a colorless solid. ^1^H NMR (400 MHz, CDCl_3_) δ: 7.48 (4H, d, *J* = 7.1 Hz), 7.29–7.41 (6H, m), 7.08 (1H, d, *J* = 2.0 Hz), 7.04 (1H, dd, *J* = 2.0, 8.3 Hz), 7.04 (1H, d, *J* = 2.0 Hz), 6.93 (1H, dd, *J* = 2.0, 8.3 Hz), 6.87 (1H, d, *J* = 16.1 Hz), 6.87 (1H, d, *J* = 8.3 Hz), 6.84 (1H, d, *J* = 8.3 Hz), 6.81 (1H, d, *J* = 16.1 Hz), 5.19 (2H, s), 5.16 (2H, s), 3.94 (3H, s), 3.89 (3H, s). ^13^C NMR (100 MHz, CDCl_3_) δ: 149.8, 149.4, 148.3, 147.8, 137.1, 137.1, 131.2, 130.6, 128.5, 128.5, 127.9, 127.8, 127.4, 127.2, 126.7, 126.6, 120.0, 119,4, 114.0, 111.9, 111.7, 109.2, 71.1, 71.0, 56.0, 56.0. IR (KBr cm^−1^): 3684, 3019, 2400, 1513, 1424, 1215, 929, 770, 669, 629, 610, 593, 580, 567, 538, 510, 470, 464, 458, 435, 420, 410, 405. EI-MS *m/z* (%): 453 (13), 452 (M+, 40), 362 (26), 361(100), 91 (47). HRMS (EI): Calcd for C_30_H_28_O_4_, 452.1988; Found: *m/z* 452.1991.

#### 4.3.13. (E)-2-(Benzyloxy)-5-(3-(benzyloxy)-4-methoxystyryl)-1,3-dimethoxybenzene (**14**)

A solution of 6 (200 mg, 0.549 mmol, 1.2 equiv.) in THF (2.8 mL) was stirred at −78 °C and added to the *t*-BuOK solution in THF (732 µL, 0.732 mmol, 1.6 equiv., 1.0 M) over 30 min. The reaction mixture was stirred for 20 min at the same temperature, and was added to 9 (125 mg, 0.457 mmol) in THF (500 µL) over 20 min and the mixture was stirred for 1 h at −78 °C and for 10 min. at 0 °C. Then, the reaction mixture was stirred for 2 h at room temperature. The reaction mixture was cooled to 0 °C and diluted with saturated NH_4_Cl solution and extracted with EtOAc (60 mL × 3), washed with saturated NH_4_Cl solution and H_2_O, dried over anhydrous Na_2_SO_4_, and concentrated. The crude product was purified over SiO_2_ column (CH_2_Cl_2_) to give 14 (81.3 mg, 37%) as a colorless solid. ^1^H NMR (400 MHz, CDCl_3_) δ: 7.27–7.51 (10H, m), 7.10 (^1^H, d, *J* = 2.0 Hz), 7.07 (1H, dd, *J* = 2.0, 8.3 Hz), 6.91 (1H, d, *J* = 16.1 Hz), 6.87 (1H, d, *J* = 8.3 Hz), 6.82 (1H, d, *J* = 16.1 Hz), 6.69 (2H, s), 5.20 (2H, s), 5.02 (2H, s), 3.90 (3H, s), 3.87 (6H, s). ^13^C NMR (100 MHz, CDCl3) δ: 153.6, 149.6, 148.3, 137.8, 137.0, 136.6, 133.3, 130.3, 128.5, 128.5, 128.1, 127.9, 127.8, 127.4, 126.8, 120.2, 111.8, 111.7, 103.4, 75.1, 71.1, 56.1, 56.0. IR (KBr cm^−1^): 3683, 3019, 2399, 1515, 1423, 1215, 1132, 928, 758, 668, 623, 530, 524, 506, 469, 419, 441, 406, 402. EI-MS *m/z* (%): 392 (28), 391 (100), 91 (21). HRMS (EI): Calcd for C_31_H_30_O_5_, 482.2093; Found: *m/z* 482.2092.

#### 4.3.14. €-4-(4-(Benzyloxy)-3,5-dimethoxystyryl)-2,6-dimethoxyphenol (**15**)

A solution of 8 (200 mg, 0.657 mmol, 1.2 equiv.) in THF (3.3 mL) was stirred at −78 °C and added to the *t*-BuOK solution in THF (1.97 mL, 1.97 mmol, 3.6 equiv., 1.0 M) over 30 min. The reaction mixture was stirred for 20 min at the same temperature, and was added to 9 (149 mg, 0.548 mmol) in THF (670 µL) over 20 min and the mixture was stirred for 1 h at −78 °C and for 10 min at 0 °C. Then, the reaction mixture was stirred for 18.5 h at room temperature. The reaction mixture was cooled to 0 °C and diluted with saturated NH_4_Cl solution and extracted with EtOAc (50 mL × 3), washed with saturated NH_4_Cl solution and H_2_O, dried over anhydrous Na_2_SO_4_, and concentrated. The crude product was purified over SiO_2_ column (n-Hex.: EtOAc = 7:3) to give 15 (49.6 mg, 21%) as a yellow solid. ^1^H NMR (400 MHz, CDCl_3_) δ: 7.50 (2H, td, *J* = 1.6, 6.6 Hz), 7.35 (2H, tt, *J* = 1.6, 6.6 Hz), 7.30 (1H, tt, *J* = 1.6, 6.6 Hz), 6.94 (1H, d, *J* = 16.2 Hz), 6.88 (1H, d, *J* = 16.2 Hz), 6.75 (2H, s), 6.71 (2H, s), 5.03 (2H, s), 3.94 (6H, s), 3.87 (6H, s). ^13^C NMR (100 MHz, CDCl3) δ: 153.6, 147.1, 137.7, 136.6, 134.7, 133.2, 128.8, 128.5, 128.2, 128.1, 127.8, 126.8, 103.3, 103.2, 75.1, 56.2, 56.1. IR (KBr cm^−1^) 3538, 3019, 2399, 1215, 756, 669, 474, 446, 430, 418, 407. EI-MS *m/z* (%): 422 (12), 332 (28), 331 (100). HRMS (EI): Calcd for C_31_H_30_O_5_, 422.1729; Found: *m/z* 422.1727.

#### 4.3.15. 5-(4-Hydroxy-3,5-dimethoxyphenethyl)-2-methoxy-3-methylphenol (RD1)

A solution of 11 (100 mg, 0.207 mmol) in THF (11 mL) was hydrogenated over 10% Pd/C (55% water, 44.3 mg) at room temperature for 19 h. The catalyst was removed by celite filtration and the filtrate was concentrated in vacuo to give RD1 (64.2 mg, quant.) as a colorless solid. ^1^H NMR (400 MHz, CDCl_3_) δ: 6.66 ^(1^H, d, *J* = 2.0 Hz), 6.52 (1H, d, *J* = 2.0 Hz), 6.38 (2H, s), 5.64 (1H, s), 5.42 (1H, s), 3.85 (6H, s), 3.77 (3H, s), 2.73–2.83 (4H, m), 2.27 (3H, s). ^13^C NMR (100 MHz, CDCl_3_) δ: 148.5, 146.8, 143.5, 138.3, 132.9, 132.8, 130.3, 122.5, 113.0, 105.0, 60.6, 56.2, 38.0, 37.8, 15.8. IR (KBr cm^−1^): 3433, 2937, 1615, 1516, 1456, 1330, 1215, 1113, 668, 467, 452, 438, 424, 414. EI-MS *m/z* (%): 318 (40), 167 (100), 151 (35). HRMS (EI): Calcd for C_18_H_22_O_5_, 318.1467; Found: *m/z* 318.1463.

#### 4.3.16. 4,4′-(Ethane-1,2-diyl)bis(2-methoxyphenol) (RD2)

A solution of 5 (50.0 mg, 0.111 mmol) in THF (6.0 mL) was hydrogenated over 10% Pd/C (55% water, 24.3 mg) at room temperature for 16 h. The catalyst was removed by celite filtration and the filtrate was concentrated in vacuo to give RD2 (30.4 mg, quant) as a colorless solid. ^1^H NMR (400 MHz, CDCl_3_) δ: 6.83 (2H, d, *J* = 8.1 Hz), 6.67 (2H, dd, *J* = 1.8, 8.1 Hz), 6.61 (2H, d, J = 1.8 Hz), 5.46 (2H, s), 3.84 (6H, s), 2.81 (4H, s). ^13^C NMR (100 MHz, CDCl_3_) δ: 146.2, 143.7, 133.7, 121.0, 114.1, 111.2, 55.8, 37.9. IR (KBr cm^−1^): 3547, 3014, 1514, 1268, 1203, 1036, 821, 675, 642, 558, 515, 506, 490, 484, 477, 462, 451, 445, 438, 429, 421, 412, 405, 401. EI-MS *m/z* (%): 274 (M+, 32), 138 (13), 137 (100). HRMS (EI): Calcd for C_16_H_18_O_4_, 274.1205; Found: *m/z* 274.1202.

#### 4.3.17. 4-(3-Hydroxy-4-methoxyphenethyl)-2-methoxyphenol (RD3)

A solution of 6 (80.0 mg, 0.177 mmol) in THF (9.3 mL) was hydrogenated over 10% Pd/C (55% water, 38.3 mg) at room temperature for 19 h. The catalyst was removed by celite filtration and the filtrate was concentrated in vacuo to give RD3 (48.5 mg, quant.) as a colorless solid. ^1^H NMR (400 MHz, CDCl_3_) δ: 6.83 (1H, d, *J* = 8.0 Hz), 6.79 (1H, d, *J* = 1.9 Hz), 6.76 (1H, d, *J* = 8.2 Hz), 6.68 (1H, dd, *J* = 1.9, 8.0 Hz), 6.64 (1H, d, *J* =1.9 Hz), 6.63 (1H, dd, J = 1.9, 8.2 Hz), 5.59 (1H, br s), 5.49 (1H, br s), 3.86 (3H, s), 3.84 (3H, s), 2.76–2.84 (4H, m). ^13^C NMR (100 MHz, CDCl_3_) δ: 146.2, 145.4, 144.7, 143.6, 135.1, 133.8), 120.9, 119.8, 114.6, 114.1, 111.0 110.5 56.0, 55.8, 37.7, 37.6. IR (KBr cm^−1^): 3545, 3019, 1513, 1272, 1215, 1035, 754, 668, 431, 419. EI-MS *m/z* (%): 274 (M+, 32), 138 (11), 137 (100). HRMS (EI): Calcd for C16H18O4, 274.1205; Found: *m/z* 274.1205.

#### 4.3.18. 4-(3-Hydroxy-4-methoxyphenethyl)-2,6-dimethoxyphenol (RD4)

A solution of 14 (50.0 mg, 0.104 mmol) in THF (5.6 mL) was hydrogenated over 10% Pd/C (55% water, 22.2 mg) at room temperature for 20 h. The catalyst was removed by celite filtration and the filtrate was concentrated in vacuo to give RD4 (31.6 mg, quant.) as a colorless solid. ^1^H NMR (400 MHz, CDCl_3_) δ: 6.79 (1H, d, *J* = 2.0 Hz), 6.76 (1H, d, *J* = 8.1 Hz), 6.62 (1H, dd, *J* = 2.0, 8.1 Hz), 6.37 (2H, s), 5.59 (1H, brs), 5.39 (1H, brs), 3.86 (3H, s), 3.85 (6H, s), 2.80 (4H, s). ^13^C NMR (100 MHz, CDCl_3_) δ: 146.8, 145.4, 144.8, 135.1, 132.9, 132.8, 119.9, 114.7, 110.6, 105.1, 56.3, 56.0, 38.2, 37.6. IR (KBr cm^−1^): 3541, 3019, 2939, 2842, 1617, 1512, 1464, 1325, 1218, 1115, 1032, 952, 759, 668, 450, 415, 404. EI-MS *m/z* (%): 304 (37), 167 (100), 137 (26). HRMS (EI): Calcd for C_17_H_29_O_5_, 304.1311; Found: *m/z* 304.1310.

#### 4.3.19. 4,4′-(Ethane-1,2-diyl)bis(2,6-dimethoxyphenol) (RD5)

A solution of 15 (23.0 mg, 0.0544 mmol) in THF (3.0 mL) was hydrogenated over 10% Pd/C (55% water, 12.1 mg) at room temperature for 21 h. The catalyst was removed by celite filtration and the filtrate was concentrated in vacuo to give RD5 (18.1 mg, quant.) as a colorless solid.^1^H NMR (400 MHz, CDCl_3_) δ: 6.36 (4H, s), 5.38 (2H, brs), 3.85 (12H, s), 2.82 (4H, s). ^13^C NMR (100 MHz, CDCl_3_) δ: 146.8, 132.9, 132.8, 105.2, 56.2, 38.5. IR (KBr cm^−1^): 3545, 3019, 2400, 1617, 1516, 1464, 1333, 1215, 1116, 760, 668, 624, 506, 498, 490, 484, 480, 475, 462, 449, 442, 438, 429, 420, 417, 405, 401. EI-MS *m/z* (%): 334 (28), 168 (16), 167 (100). HRMS (EI): Calcd for C1_8_H_22_O_6_, 334.1416; Found: *m/z* 334.1415

### 4.4. Preparation of the Resveratrol Derivatives Stock Solution

Resveratrol derivatives (RD1-RD5) were prepared as a 40 mM master stock solution by dissolving in DMSO solution and stored at −20 °C. The compounds were freshly diluted with medium to the desired concentrations before using. The final concentration of DMSO in solution was less than 0.5%, which caused no signs of cytotoxicity. 

### 4.5. Cell Viability

Cell viability was investigated by MTT colorimetric assay. All cell lines were cultured at the density of 1 × 10^4^ cells per well in a 96-well tissue culture plate and incubated in an incubator. After that, cells were treated with various concentrations of RD1-RD5 for 24 h at 37 °C and analyzed by the MTT assay according to the manufacturer’s protocol (Sigma Chemical, St. Louis, MO, USA). In calculating the cell viability by a microplate reader (Anthros, Durham, NC, USA), the measured absorbance of treated cells were divided by the value of untreated cells and are reported as a percentage.

### 4.6. Nuclear Staining Assay

This method was applied to define apoptotic and necrotic cell death by using nuclear staining with Hoechst 33342. The cells were seeded on 96-well plates at the density 1 × 10^4^ cells per well in 96-well plates and incubated overnight. The cells were treated with various concentrations of RD1-RD5 and incubated for 24 h at 37 °C. Afterward, the cells were incubated with Hoechst 33342 (10 μg/mL) / PI (5 μg/mL) solution for 30 min at 37 °C. Then, they were visualized and imaged under a fluorescence microscope (Olympus DP70, Melville, NY, USA). Results are reported as a percentage of apoptotic cells.

### 4.7. Colony Formation Assay

The measurement of clonogenicity was demonstrated by colony formation assay. Cells were seeded at a density of 300 cells/well into 6-well plate and then further treated with various concentrations resveratrol derivatives for 24 h after that the medium was replaced and the cell were incubated for colony number and colony size were investigated after fixing with methanol and acetic acid (3:1) solution and staining with 0.05% *w*/*v* crystal violet in 4% formaldehyde.

### 4.8. Western Blot Analysis

Western blotting was used to detect the presence of specific protein. Cells were seed at a density of 4 × 10^5^ cells/well in 6 well plates overnight. Cells were treated with various concentrations RD1-RD5 were treated for 12 h. After treatment, the apoptosis cells were collected by centrifuging media with 1500 rpm for 5 min and aspirating supernatants. Then, cells were washed with cold 1× PBS and incubated with 1X RIPPA 40 µL containing 10× RIPA buffer 100 µL, protease inhibitors (PI) 100 µL, PMSF 10 µL, and Triton × 10 µL for 30 min on ice. BCA protein assay kit from Pierce Biotechnology (Rockford, IL, USA) analyzed protein concentrations. The extracted proteins were separated by 7.5–10% sodium dodecyl sulfate polyacrylamide gel electrophoresis (SDS-PAGE) and transferred to polyvinylidene difluoride membrane (PVDF). Then, the membranes were blocked with 5% non-fat milk powder for 2 h. After that, they were incubated with primary antibody at 4 °C Akt (#9272), phosphorylated Akt (#4060), mTOR (#2983S), and *p*-mTOR (#5536S) overnight. The membranes were washed with Tris-buffered saline/Tween 20 3 times and incubated with secondary antibodies for 2 h. Finally, the protein bands were detected using chemiluminescence substrate and exposed by Chemiluminescent ImageQuant LAS4000. Protein bands were analyzed using Image J software.

### 4.9. Immunofluorescence Assay

Cells were seeded at a density of 1 × 10^4^ cells/well in 6-well plates overnight. Cells were treated with various concentrations resveratrol derivatives (0–100 µL) and were treated for 12 h. Then, they were fixed with 4% paraformaldehyde in PBS for 15 min, permeabilized for 5 min with 0.5% Triton-X, and blocked with 0.1% Triton-X in 10% FBD in PBS for 1 h at room temperature. Cells were added to the primary antibody Akt (#9272), phosphorylated Akt (#4060), mTOR (#2983S), and *p*-mTOR (#5536S). Then, they were incubated at 4 °C overnight. After that, they were incubated with secondary antibody for 1 h and stained with Hoechst 33342 for 30 min at room temperature in a dark place. The images were visualized under a fluorescent microscope (Olympus DP70, Melville, NY, USA) and analyzed by The ImageJ software.

### 4.10. Molecular Docking 

The protein structures of Akt-1 with the ATP-competitive inhibitor (PDB ID 3CQW) [37] and the allosteric inhibitor (PDB ID 5KCV) [38] and the PH domain of Akt-1 (PDB ID 1UNQ) [39] were received from the Research Collaboratory for Structural Bioinformatics Protein Data Bank [40]. All protein structures were prepared for docking using UCSF Chimera version 1.16 [41]. The AutoDockTools (ADT) suite [42] was used to add the polar hydrogen atoms and convert them into the PDBQT files.

The 3D structures of the three ligands were obtained from the ZINC15 database [43] (ZINC6787, ZINC196360119, and ZINC52650381 for resveratrol (RS) and RD2, respectively. The 3D structure of SC79 was obtained from the PubChem database [44]. The ligands were optimized using the B3LYP/6-31G (d, *p*) basis set implemented in the Gaussian 09 (G09) program [45] and converted into PDBQT files using the ADT.

Ligands were docked at the Akt-1 using the AutoDock Vina 1.2.3 program [46] with a Vina forcefield to investigate the binding affinity and mode at the atomic level. The binding pocket was determined based on the position of the co-crystal ligand. Grid box was set to 20 Å × 20 Å × 20 Å with grid center defined at x = 5.274, y = 2.843, z = 18.247 (PDB ID 3CQW) and x = −9.843, y = −1.517, z = −17.927 (PDB ID 5KCV)), and x = 15.18, y = 24.427, z = 16.345 (PDB ID 1UNQ). The UCSF ChimeraX version 1.3 was used to visualize and analyze the key binding modes.

### 4.11. Statistical Analysis

All results were compared and expressed as mean ± standard error of the mean (SEM) from at least triplicate independent experiments. Statistical analyses were evaluated using analysis of variance (ANOVA). The statistic was calculated by using SPSS version 28 (IBM Corp., Armonk, NY, USA). Statistically significant differences were indicated by * *p*-values less than 0.05. GraphPad Prism 9 was used for creating graphs in all experiments (GraphPad Software, San Diego, CA, USA).

## 5. Conclusions

In conclusion, these results provide novel and significant data on the resveratrol derivatives. 4,4′-(ethane-1,2-diyl)bis(2-methoxyphenol) (RD2) can be considered as a potential therapy for NSCLC through the suppression of Akt/mTOR-mediated survival. In addition, RD2 could exert the inhibition of the ATP-binding site and allosteric binding site with the Akt protein. As Akt is critical for cell survival, our results might be used in demonstrating that RD2 may represent a potential therapeutic candidate for the treatment of lung cancer. 

## Figures and Tables

**Table 1:** The binding affinities of ligands docked with the ATP-binding site of Akt-1.

Compound	Binding Affinity (Vina) (kcal/mol)	Hydrogen Bond Interaction
Resveratrol	−8.054	Ala230
RD2	−8.041	Glu228, Ala230, Asp292
CQW (reference compound)	−8.346	Glu228, Ala230, Glu234

**Table 2:** The binding affinities of ligands docked with the allosteric site of Akt-1.

Compound	Binding Affinity (Vina) (kcal/mol)	Hydrogen Bond Interaction
Resveratrol	−8.446	Asn54, Thr82, Gln203, Ser205
RD2	−8.546	Asn54, Gln79 (2 bonds), Ser205
CQW (reference compound)	−12.344	Thr211 (2 bonds), Tyr272, Asp274

**Table 3:** The binding affinities of ligands docked with the PH domain of Akt-1.

Compound	Binding Affinity (Vina) (kcal/mol)	Hydrogen Bond Interaction
RD2	−5.297	Gln79
RD3	−6.072	Ile19, Arg23, Arg25
SC79 (Akt activator)	−5.620	Lys14, Tyr18, Ile19, Arg23
IP4 (reference compound)	−7.209	Lys14, Glu17, Tyr18, Ile19, Arg23, Arg25, Arg86

## Data Availability

Data are contained within the article.
